# Production of thermostable phycocyanin in a mesophilic cyanobacterium

**DOI:** 10.1016/j.mec.2021.e00175

**Published:** 2021-06-02

**Authors:** Anton Puzorjov, Katherine E. Dunn, Alistair J. McCormick

**Affiliations:** a SynthSys & Institute of Molecular Plant Sciences, School of Biological Sciences, University of Edinburgh, Edinburgh, EH9 3BF, UK; b Institute for Bioengineering, School of Engineering, University of Edinburgh, Edinburgh, EH9 3DW, UK

**Keywords:** Allophycocyanin, Energy transfer, Linker, Phycobilisome, *Synechocystis* sp. 6803, *Thermosynechococcus elongatus* BP-1

## Abstract

Phycocyanin (PC) is a soluble phycobiliprotein found within the light-harvesting phycobilisome complex of cyanobacteria and red algae, and is considered a high-value product due to its brilliant blue colour and fluorescent properties. However, commercially available PC has a relatively low temperature stability. Thermophilic species produce more thermostable variants of PC, but are challenging and energetically expensive to cultivate. Here, we show that the PC operon from the thermophilic cyanobacterium *Thermosynechococcus elongatus* BP-1 (*cpcBACD*) is functional in the mesophile *Synechocystis* sp. PCC 6803. Expression of *cpcBACD* in an ‘Olive’ mutant strain of *Synechocystis* lacking endogenous PC resulted in high yields of thermostable PC (112 ± 1 mg g^−1^ DW) comparable to that of endogenous PC in wild-type cells. Heterologous PC also improved the growth of the Olive mutant, which was further supported by evidence of a functional interaction with the endogenous allophycocyanin core of the phycobilisome complex. The thermostability properties of the heterologous PC were comparable to those of PC from *T. elongatus*, and could be purified from the Olive mutant using a low-cost heat treatment method. Finally, we developed a scalable model to calculate the energetic benefits of producing PC from *T. elongatus* in *Synechocystis* cultures. Our model showed that the higher yields and lower cultivation temperatures of *Synechocystis* resulted in a 3.5-fold increase in energy efficiency compared to *T. elongatus*, indicating that producing thermostable PC in non-native hosts is a cost-effective strategy for scaling to commercial production.

## Highlights

• *Synechocystis* sp. PCC 6803 was used as a platform to produce thermostable phycocyanin.
• Phycocyanin from *Thermosynechococcus elongatus* BP-1 was expressed at native levels.
• Heterologous phycocyanin could transfer energy to the allophycocyanin core *in vivo.*
• A low-cost heat treatment was developed to purify heterologous phycocyanin.
• Production of thermotolerant phycocyanin in *Synechocystis* is more energy efficient.

## 1 Introduction

Phycocyanin (PC) is a blue pigment-protein (i.e. a phycobiliprotein) that forms part of the photosynthetic light-harvesting phycobilisome (PBS) complex in most cyanobacteria and red algae. PC is considered a high-value product that is in significant demand from the food and cosmetics industries, as it is currently the only commercially available naturally-derived, soluble blue colourant (Kupferschmidt, 2019; Nowruzi et al., 2020). PC is also a potent fluorophore with anti-oxidant properties, and thus is of interest to several other market sectors, including biopharmaceuticals, nutraceuticals, and research diagnostics (Eriksen, 2008; Li et al., 2019; Pagels et al., 2019). In addition, PC has shown promise in biophotovoltaic applications (e.g. as a natural photosensitizer for dye-sensitised solar cells) (Hartmann et al., 2020).

Nevertheless, the commercial applications of PC are currently limited to specific applications in the food industry due to its narrow temperature stability range (Puzorjov and McCormick, 2020). PC is harvested primarily from the mesophilic cyanobacterium *Arthrospira platensis* (a GRAS-approved species, commonly known as Spirulina), which grows optimally at 20–35 °C (Colla et al., 2007). Exposure of *A. platensis* PC to temperatures exceeding 45 °C results in progressive protein denaturation, loss of colour and a reduction in anti-oxidant properties, which limit its usage in industries including food, cosmetics and textiles where processing temperatures often exceed 45 °C (Chaiklahan et al., 2012; Jespersen et al., 2005; Ogbonda et al., 2007; Pan-utai et al., 2018; Sarada et al., 1999). Additives and/or cross-linking agents can improve the stability of *A. platensis* PC (Chaiklahan et al., 2012; Martelli et al., 2014; Pan-utai et al., 2018). However, additives can increase the cost of the final product and affect colour and taste, while cross-linking agents (e.g. formaldehyde) are often too toxic for human consumption (Azeredo and Waldron, 2016).

Thermophilic species, such as *Thermosynechococcus* spp*.* and *Galdieria* spp*.*, do produce a more thermostable form of PC compared to *A. platensis* (Ferraro et al., 2020a; Liang et al., 2018; Moon et al., 2014). As of 2021, at least two companies (i.e. Fermentalg and Matis) are cultivating thermophilic strains for PC extraction. However, the energetic costs of maintaining and culturing thermophiles at optimal growth temperatures is significantly higher compared to mesophilic species (Huang et al., 2017). Furthermore, the maximum growth rates of thermophiles as well as the PC content are typically lower (Klepacz-Smolka et al., 2020; Liang et al., 2018; Miller and Castenholz, 2000). As a result, the highest reported productivity for a thermostable PC from cyanobacteria (*Synechococcus lividus* PCC 6715) grown in optimised blue light conditions is ~23 mg L^−1^ d^−1^ (Klepacz-Smolka et al., 2020; Liang et al., 2018). In comparison, the levels of PC productivity from *A. platensis* can reach up to 125 mg L^−1^ d^−1^ (Chen et al., 2013a).

Heterologous expression of individual α- or β-subunits of phycobiliproteins (PBPs) has been demonstrated in *Escherichia coli* (Alvey et al., 2011a; Chen et al., 2013b, 2016; Tooley et al., 2001). However, production of mature PC is significantly more challenging, and requires expression of up to nine genes, including both ⍺- and β-subunits (*cpcA* and *cpcB*), five lyase components (*cpcE, cpcF*, *cpcS*, *cpcU* and *cpcT*) and genes required to convert heme into the phycocyanobilin (PCB) chromophore (*ho1* and *pcyA*) (Bolte et al., 2008; Chen et al., 2013b; Fairchild et al., 1992; Shen et al., 2008; Tooley et al., 2001; Zhao et al., 2017). Furthermore, partial chromophorylation of individial PBP subunits remains a key metabolic engineering challenge in *E. coli* (Chen and Jiang, 2019; Chen et al., 2020; Zhao et al., 2007), and to date no studies have reported heterologous expression of mature PC.

An alternative strategy would be to engineer an efficient mesophilic cyanobacterial host for expression of heterologous PC variants with superior properties. Cyanobacterial mutant strains lacking endogenous PC have a distinctive pale green phenotype and typically grow slower than wild-type (WT) under light limited conditions (Kirst et al., 2014; Lea-Smith et al., 2014). PC-deficient mutants have been studied in a variety of cyanobacterial species including *Synechocystis* (Elmorjani et al., 1986; Liberton et al., 2017; Plank et al., 1995; Vasudevan et al., 2019), *Synechocystis* sp. PCC 6701 (Anderson and Grossman, 1990), *Synechocystis* sp. PCC 6714 (Nakajima et al., 1998; 2001), and filamentous *Anabaena* sp. PCC 7120 (Leganes et al., 2014). Leganes et al. (2014) also demonstrated that the WT phenotype of a PC-deficient *Anabaena* sp. PCC 7120 mutant could be restored by introduction of a self-replicating plasmid carrying an expression cassette for the complete endogenous PC operon.

Here, we have expressed variants of the PC operon from *T. elongatus* (*cpcBACD*) in a PC-deficient ‘Olive’ mutant of *Synechocystis* lacking the endogenous PC operon (*cpcBAC2C1D*) (Vasudevan et al., 2019). Remarkably, we observed heterologous *T. elongatus* PC production similar to native PC levels in WT *Synechocystis*. The proteins encoded by *cpcBAC2C1D* and *cpcBACD* are adapted to function optimally at significantly different temperatures (i.e. the optimal growth for *Synechocystis* is typically 30 °C compared to 45 °C for *T. elongatus*) (Inoue et al., 2001; Liang et al., 2018). Nevertheless, we observed that expression of *T. elongatus* PC partially complemented the growth of the Olive mutant, and we found evidence of interaction between the thermostable PC and the endogenous allophycocyanin (APC) core *in vivo*. We evaluated PC and APC content and biomass productivity of the Olive mutants complemented with variants of the *T. elongatus* PC operon, as well as the energetic costs of growing complemented Olive mutants at 30 °C. We also examined the stability of the heterologous PC and have established a simple and low-cost, heat treatment method to purify thermostable PC from soluble native contaminants, including APC.

## 2 Materials and methods

### 2.1 Plasmid construction

All molecular cloning was performed using CyanoGate, a Golden Gate MoClo toolkit for cyanobacteria, which is compatible with the plant MoClo toolkit (Engler et al., 2014; Vasudevan et al., 2019). Previously created parts and acceptor vectors were used derived from CyanoGate (pC) and the plant MoClo toolkit (pICH). Primers used in this study were synthesised by Integrated DNA Technologies (IDT) (Supplementary Table S1). Variants of the PC operon were PCR amplified from the wild-type *Synechocystis* and *T. elongatus* genomic DNA using Q5 High-Fidelity DNA Polymerase (New England Biolabs) and assembled into Level 0 CDS acceptor vector (pICH41308). Parts were domesticated (*Bsa*I and *Bpi*I recognition sites removed) as necessary using a method described previously (Gale et al., 2019). Native PC operon promoter (P_cpc560_, pC0.005) and terminator (T_cpc_, pC0.078) from *Synechocystis* were cloned upstream and downstream of all PC operon variants into Level 1 Position 2 (reverse) acceptor vector (pICH47811) (Vasudevan et al., 2019). All Level 1 PC operon variants were assembled into the self-replicative pPMQAK1-T (pCAT.000) acceptor vector using a Level 1 Position 1 “Dummy” (pICH54011) and End-Link 2 (pICH50881) parts (Gale et al., 2019; Vasudevan et al., 2019). All expression vectors were conjugated into an unmarked (i.e. lacking an antibiotic selection cassette) *Synechocystis* ‘Olive’ mutant (Δ*cpcBAC2C1D*) (SynO) via triparental mating, as described in Gale et al. (2019).

### 2.2 Culture conditions

Liquid cultures of *Synechocystis* and *T. elongatus* were grown in BG11 medium at 30 °C and 50 °C, respectively, under continuous white LED light (50 μmol photons m^−2^s^−1^) in an Infors Multitron-Pro (Infors HT) incubator shaken at 100 rpm and aerated with ﬁlter sterilised water-saturated atmospheric air. To minimise evaporation from the liquid cultures at high incubation temperatures, the humidity levels inside incubator was maintained at 95% using an external air humidifier (Taotronics TT-AH001). Transconjugated strains were grown in BG11 medium supplemented with 50 μg mL^−1^ kanamycin. Growth experiments were performed with four biological replicates. Whole-cell absorption spectra (400–750 nm) of cyanobacterial cultures were measured in 96-well flat-bottom (Chimney Well) μCLEAR plates using a FLUOstar OMEGA microplate reader (BMG Labtech). OD_750_ was measured using Biochrom WPA Biowave II Spectrophotometer.

### 2.3 Measurement of chlorophyll content

Cell cultures were diluted to OD_750_ = 1.0 and 1 mL of culture was centrifuged at 17,000 *g* for 2 min. The pellet was resuspended in 100% methanol and shaken at 2400 rpm for 1 h in the dark at room temperature using an IKA-VIBRAX-VXR bead beater. The cells were centrifuged at 17,000 *g* for 10 min and the absorbance of the supernatant was measured at 652, 665 and 750 nm. The mean concentration of Chl *a* was calculated from triplicates as described in Porra et al. (1989).

### 2.4 Extraction of phycobiliproteins and phycobilisomes

PC and APC were extracted and quantified using absorbance spectroscopy as described previously (Bennett and Bogorad, 1973; Zavrel et al., 2018). To extract the PBSs, cyanobacterial cells were pelleted by centrifugation at 4000 *g* for 15 min, washed in 0.8 M phosphate buffer (pH 7.0) three times and resuspended in 1 mL of fresh phosphate buffer. Cells were lysed with 0.5 mm glass beads (BioSpec Products) on a VXR Vibrax orbital shaker (IKA) shaking at 2400 rpm for 30 min at 4 °C in the dark. Triton X-100 (Sigma-Aldrich) was added to a final concentration of 2% (v/v) and samples were shaken at 200 rpm for 30 min at room temperature in the dark (Kondo et al., 2005). Following centrifugation for 30 min at 20 °C at 17,000 *g*, the aqueous blue liquid layer containing PBSs was extracted and PC and APC quantified using a FLUOstar OMEGA microplate reader (BMG Labtech).

### 2.5 SDS-PAGE and zinc-induced fluorescence assay

Samples equivalent to OD_620_ = 0.5 were analysed on NuPAGE 12% Bis-Tris (Invitrogen) protein gel set to run at 150 V for 1 h. SeeBlue Plus2 Pre-Stained (Invitrogen) protein standard was used as a ladder. The gels were incubated in 100 mM zinc sulfate solution for 10 min and visualized under UV light for the presence of zinc-induced fluorescence of bilin chromophores (Raps, 1990). The gels were further stained with 1% (v/v) Coomassie Blue in acetic acid/methanol. For the bands extracted and purified from sucrose gradients (section 2.9) Coomassie staining was not able to clearly visualize the bands. Therefore, the gel was re-stained using silver staining as described in Heukeshoven and Dernick (1985).

### 2.6 Biomass and phycocyanin productivities

The volumetric biomass productivity was (*P*, mg L^−1^ d^−1^) was calculated using the following equation (Zhang et al., 2015):

$$P\;=\;\frac{W_{n}-\;W_{0}}{n}$$

where *W*_*0*_ and *W*_*n*_ are the biomass concentrations (mg L^−1^) at the start and at the end of the exponential stage, respectively, and *n* is the number of days between measurements. The PC productivity was then calculated by multiplying the PC content (g g^−1^ DW) by the biomass productivity of each strain.

### 2.7 Thermal stability of phycocyanin *in vitro*

Following the extraction, PC containing solutions were diluted to 0.1 g mL^−1^. Fluorescence was measured in a far-UV quartz cuvette (10 mm path length) filled with 3 mL of PC solution using a Cary Eclipse Fluorescence Spectrophotometer (Agilent) equipped with Multicell Holder. The solutions were excited at 605 nm and emission of PC was measured at 650 nm. Fluorescence was measured starting from 25 °C and then every 30 s as the sample was heated up to 75 °C at a rate of 0.1 °C per sec. The change in fluorescence was calculated relative to the initial fluorescence at 25 °C. PC solutions were stirred with a magnetic stir bar, and the temperature inside the cuvette was monitored using a built-in probe. Measurements of PC extracted from each strain were taken in triplicate. The first derivative was calculated for each replicate and a Gaussian was fitted to find the temperature at which degradation reaches the maximal value.

### 2.8 Heat treatment purification of thermostable phycocyanin

PC extracts were diluted to OD_620_ = 1.0 and incubated at 60 °C for 15 min on a heating block. Immediately after incubation, the tubes were cooled down on ice. Both heat-treated and untreated controls were centrifuged for 8 min at 17,000 *g* and 25 μL of the supernatant was used for SDS-PAGE analysis.

### 2.9 Sucrose gradients

A sample containing ~300 μg of PBS was loaded onto sucrose step gradient prepared by layering from bottom upward: 2 mL of 2.0 M, 3 mL of 1.0 M, 2.5 mL of 0.75 M, 2.5 mL of 0.5 M, and 2 mL of 0.25 M sucrose solutions in 0.8 M PB, pH 7.0, in 14 mL Ultra-Clear centrifuge tubes (Beckman, cat. 344060). The resulting gradients were centrifuged in a Beckman SW 40 Ti Swinging-Bucket Rotor at 220,000 *g* (40,000 rpm) for 18 h at 20 °C. Blue-colored fractions were collected from the sucrose gradients using a syringe. The sucrose in each fraction was diluted with 5 vol of PB before proteins in each fraction were precipitated by adding ammonium sulfate to 50% (w/v) (Safaei et al., 2019; Su et al., 1992). Resulting pellet was resuspended in 15 μL of PB and used for analysis on SDS-PAGE gel.

### 2.10 Thermal stability of phycobilisomes and PSII *in vivo*

Samples were diluted to the same cell density (OD_750_ = 1.0, Chl = 3.0 μg mL^−1^) and dark adapted for 15 min prior to taking measurements. Measurements were done using Agilent Cary Eclipse Fluorescence Spectrophotometer equipped with Ambient Multicell Holder. Cells were excited either at 437 or 605 nm to excite PSII or PBS respectively. Fluorescence was measured starting from 25 °C and then every 30 s as the sample was heated up to 75 °C at a rate of 0.1 °C per sec. Cells were stirred with a magnetic stir bar, and the temperature inside the cuvette was monitored using a built-in probe. Measurements for each strain were taken in technical triplicates.

### 2.11 Whole-cell absorption and fluorescence measurements

Whole-cell absorption spectra (400–750 nm) of cyanobacterial cultures were measured in 96-well flat-bottom (Chimney Well) μCLEAR plates using a FLUOstar OMEGA microplate reader (BMG Labtech). OD_750_ was measured using Biochrom WPA Biowave II Spectrophotometer. For measuring whole-cell fluorescence at room temperature, samples of OD_750_ = 2.0 were diluted to the same cell density (OD_750_ = 1.0, Chl = 3.0 μg mL^−1^) and dark adapted for 15 min. Fluorescence emission was measured in a far-UV quartz cuvette (10 mm pathlength) filled with 3 mL of cell culture using a Cary Eclipse fluorescence spectrophotometer. Excitation wavelengths of 435 nm and 580 nm were used to excite chlorophyll and PBS components, respectively. The emission spectrum was measured from 600 to 800 nm. All emission spectra were normalised to 684 nm for chlorophyll and to the max peak for PBS (645–662 nm).

For measuring whole-cell fluorescence at 77 K, samples were diluted to the same cell density (OD_750_ = 2.0, Chl = 6.0 μg mL^−1^) and dark adapted for 15 min. Fluorescence spectrum was read using HORIBA FluoroMax-P Samples in custom made quartz tubes (diameter = 6.0 mm) were placed in a FL-1013 liquid nitrogen dewar assembly (Horiba). Fluorescence emissions were measured using a FluoroMax-P spectrofluorometer (Horiba) with excitation/emission wavelengths as above. Emission spectra were normalised to 684 nm for chlorophyll and to 800 nm for PBS.

## 3 Results and discussion

### 3.1 Overview of phycobilisomes in *Synechocystis* and *Thermosynechococcus*

**Fig. 1:**
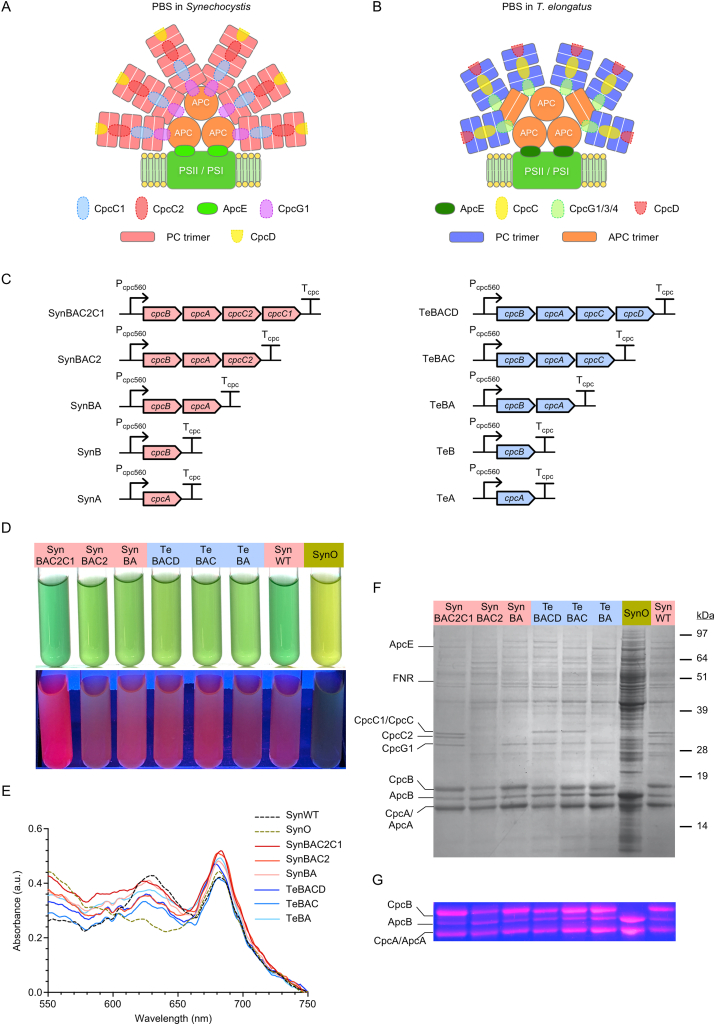
Characterization of transconjugant *Synechocystis* Olive mutants expressing endogenous or heterologous phycocyanin. (A) Schematic structure of the phycobilisome complex in *Synechocystis* sp. PCC 6803 (Synechocystis) (Liu et al., 2021) and (B) in *Thermosynechococcus elongatus* (Chang et al., 2015; David et al., 2014). (C) Expression cassettes encode genes from the phycocyanin (PC) operon of *Synechocystis* (red) or *T. elongatus* (blue) were driven by the native PC operon promoter (P_cpc560_) and terminator (T_cpc_). (for full sequences see Supplementary Information S1) (D) Transconjugated strains in ambient light (top) and UV light (312 nm) (bottom). (E) Whole-cell absorption spectra with the absorption band for PC visible in the 620–630 nm region. (F) SDS-PAGE gel of PC extracts. Peptides were visualized with Coomassie brilliant blue staining. (G) Zinc-induced fluorescence of chromophorylated phycobiliproteins under UV light. Abbreviations: ApcA/ApcB, allophycocyanin ⍺- and β-subunits; ApcE, allophycocyanin core linker; CpcC/CpcC1/CpcC2, rod linker proteins; CpcG1/CpcG2/CpcG4, rod-core linker proteins; CpcA/CpcB, phycocyanin ⍺- and β-subunits; CpcD, rod capping linker; FNR, ferredoxin-NADP^+^ oxidoreductase; SynO, *Synechocystis* Olive mutant; SynWT, *Synechocystis* (wild-type); Te, *T. elongatus*. (For interpretation of the references to colour in this figure legend, the reader is referred to the Web version of this article.)

PC consists of two PBP ⍺- and β-subunits that assemble into a stable heterodimeric (⍺β) monomer. The (⍺β) monomer is decorated with three linear PCB chromophores that are covalently attached to each subunit by specific PBP lyases at well-conserved cysteine residues (i.e. ⍺-Cys-84, β-Cys-84 and β-Cys-155). Three (αβ) monomers then self-assemble to form a trimeric (αβ)_3_ disk, and two (αβ)_3_ trimers aggregate to form a dual-disk (αβ)_6_ hexamer. Similarly to *A. platensis*, the PBS in the model mesophilic cyanobacterium *Synechocystis* sp. PCC 6803 (hereafter *Synechocystis*) is made of two domains and typically associates with Photosystem II (PSII): i) a tricylindrical APC core and ii) six PC rods, each consisting of three (αβ)_6_ hexamers, emanating from the core (Fig. 1A) (Liu et al., 2021). The APC core is attached to the stromal-facing surface of the thylakoid-embedded PSII complex via the core-membrane linker ApcE (120 kDa) (Chang et al., 2015; Ma et al., 2020; Zhang et al., 2017). The three (αβ)_6_ hexamers are linked by the rod-core linker CpcG1 (28.9 kDa) and rod linker proteins, CpcC1 (33 kDa) and CpcC2 (31 kDa) located at the proximal, middle and distal end of the rod from the APC core, respectively (Lee et al., 2019; Niedzwiedzki et al., 2019). A small capping rod linker CpcD (9 kDa) is thought to stabilise the rod structure by binding at the core-distal end of each rod (de Lorimier et al., 1990). An alternative form of the PBS complex has also been observed that consists of a single elongated rod made of up to five (αβ)_6_ PC hexamers linked by one CpcC1 and several CpcC2 linkers (Liu et al., 2019; Watanabe et al., 2014). The latter PBS complex appears to preferentially associate with Photosystem I (PSI) via the linker CpcL (28.5 kDa) (previously named CpcG2) and does not have an APC core. In contrast to *Synechocystis*, the PBS in *Thermosynechococcus* spp. appears to have a pentacylindrical APC core and 6–8 PC rods consisting of two (αβ)_6_ hexamers each (Fig. 1B). Each rod is linked by the rod-core linker CpcG1/2/4 (29–31 kD) and one rod linker CpcC (33 kD) (Chang et al., 2015; David et al., 2014). CpcL appears absent in *Thermosynechococcus* spp., suggesting that this genus may not assemble rod-shaped PBS complexes (Hirose et al., 2019).

To the best of our knowledge, only one previous study has reported on the expression of heterologous PC in a cyanobacterium (Plank and Anderson, 1995). Plank and Anderson (1995) expressed a partial PC operon (*cpcBA*) in *Synechocystis* from the closely related species PCC 6701 (i.e. PC ⍺- and β-subunits from PCC 6701 share 82.2% and 81.4% protein sequence identity, respectively, to the endogenous *Synechocystis* isoforms). The heterologous PC subunits assembled with the endogenous rod linkers (CpcC1, CpcC2), the rod-core linker (CpcG1) and the APC core, and produced a functional PBS pool, albeit with a decreased average rod length due to a reduced incorporation of CpcC2 compared to that of the WT, but with a similar capacity to transfer the excitation energy to PSII. Further work has demonstrated that, in some cases, native and heterologous PBS components can be recombined *in vitro* to form a functional PBS complex (e.g. the APC core and rods isolated *Nostoc* sp., *Synechococcus* sp. PCC 7942 and *Fremyella diplosiphon*) (Canaani and Gantt, 1982; Canaani et al., 1980; Glick and Zilinskas, 1982; Khanna et al., 1986). More recent work has indicated that the assembly of the PBS does not need to be structurally homogenous for efficient energy transfer (David et al., 2014), which suggests that even partial compatibility between native and heterologous PBS components could facilitate energy transfer *in vivo*.

### 3.2 Expression of endogenous or heterologous *cpcA* and *cpcB* resulted in PC production in the *Synechocystis* Olive mutant

Ten self-replicating vectors carrying expression cassettes with variants of the PC operon from *Synechocystis* or *T. elongatus* were transconjugated into a previously generated *Synechocystis* ‘Olive’ mutant strain (SynO) (Vasudevan et al., 2019). The resulting ten transconjugant strains were named based on the expression vectors carrying variants of the *Synechocystis* PC operon (i.e. SynBAC2C1, SynBAC2, SynBA, SynB and SynA) or *T. elongatus* PC operon (i.e. TeBACD, TeBAC, TeBA, TeB and TeA) (Fig. 1C).

Following transconjugation, colonies developed for all ten strains on BG11 agar plates supplemented with kanamycin, and all strains grew in BG11-medium containing kanamycin. The four strains carrying expression vectors with only a single PC subunit (i.e. *cpcA* or *cpcB*) were similar to SynO and did not show a characteristic red fluorescence signal for PC under UV light (Seppälä et al., 2007) (Supplementary Fig. S1A). Furthermore, cell extracts from these strains lacked a characteristic peak at 620 nm for PC (Supplementary Fig. S1B), suggesting that the single subunits of PC were not expressed. Previous work has indicated that expression of both subunits is required to generate a stable (⍺β) monomer, without which either subunit is rapidly degraded (Plank and Anderson, 1995; Plank et al., 1995). Thus, these four strains were excluded from further analysis.

The remaining six strains carried expression vectors with both *cpcA* and *cpcB* (Fig. 1C). Remarkably, all six strains, including those with the genes from *T. elongatus*, were visibly less pale than SynO and showed a blue-green phenotype similar to WT (Fig. 1D). Furthermore, a clear red fluorescence signal characteristic of PC (Seppälä et al., 2007) was observed in these strains under UV light. Whole-cell absorbance spectroscopy showed an additional band in the 620–630 nm region corresponding to the maximum absorbance of PC, which was absent in SynO (Fig. 1E). Thus, our data indicated that both endogenous PC and heterologous PC from *T. elongatus* (Te-PC) could be produced in the SynO host.

Analysis of soluble protein extracts by SDS-PAGE showed the presence of bands corresponding to PC ⍺- and β-subunits in the transconjugant strains, and their absence in SynO (Fig. 1F). Fluorescence of these bands following incubation with zinc sulfate indicated that both PC subunits were chromophorylated with phycocyanobilins (Fig. 1G). Specifically, the fluorescence observed for CpcA and CpcB bands in TeBA, TeBAC and TeBACD indicated that the endogenous lyases (i.e. CpcE/F, CpcU/S and CpcT) in *Synechocystis* were able to attach bilin chromophores to the ⍺- and β-subunits of Te-PC. The ability of cyanobacterial lyases to attach both endogenous and heterologous bilin chromophores to various PBP subunits has been previously demonstrated *in vitro* (Alvey et al., 2011a, 2011b; Chen et al., 2013b). Furthermore, Chen et al. (2013b; 2016) showed in *E. coli* that CpcU/S from *Synechocystis* were able to chromophorylate the ⍺- and β-subunits of APC from *T. elongatus*. Our results provide further evidence of the promiscuity of the PBP lyases *in vivo*.

Rod linker proteins CpcC2 (31 kDa) and CpcC1 (33 kDa) were both successfully expressed in SynBAC2C1 (Fig. 1F). However, CpcC2 was absent in SynBAC2, which carried the gene for CpcC2 but not CpcC1. Previous work has demonstrated that disruption of *cpcC1* results in the loss of expression of both CpcC1 and CpcC2 (Ughy and Ajlani, 2004). In contrast, when *cpcC2* was disrupted, CpcC1 was still expressed. Thus, CpcC1 may have an epistatic influence on CpcC2 expression. Our results for SynBAC2 are consistent with those findings. Notably, the rod-core linker protein CpcG1 (29 kDa) also appeared absent in SynBAC2 (in contrast to previous work in Δ*cpcC1*) (Ughy and Ajlani, 2004), but was present in all other complemented lines, including TeBACD, TeBAC and TeBA. Due to the very similar protein profile of SynBAC2 to SynBA (barring the absence of CpcG1), the former strain was excluded from further analysis.

The heterologous rod linker protein CpcC (33 kDa) from *T. elongatus* was expressed in both TeBACD and TeBAC strains. Both CpcC and CpcC1 connect the first two PC hexamers within the rod to the APC core (David et al., 2011; Guan et al., 2007; Ughy and Ajlani, 2004). A phylogenetic comparison showed that CpcC is more closely related to CpcC1 than CpcC2 (Supplementary Fig. S2), which may explain why CpcC was not degraded in *Synechocystis*.

### 3.3 Olive mutants were complemented by Te-PC and had improved growth rates

**Fig. 2:**
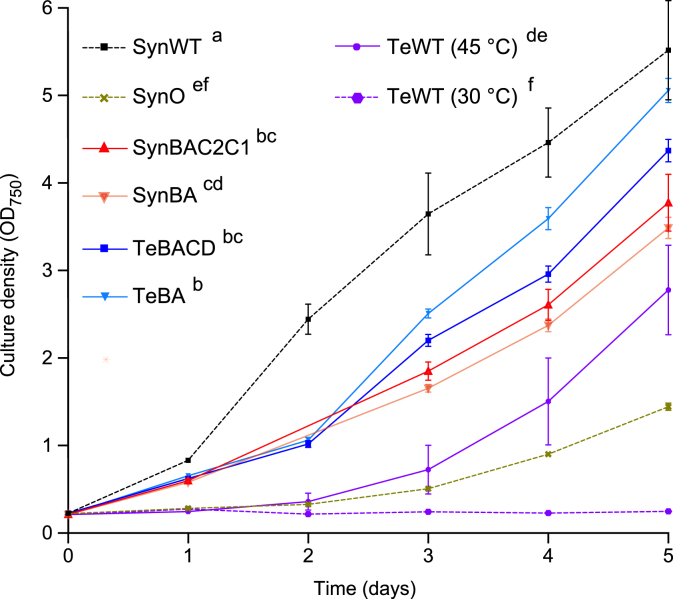
Growth analysis of wild-type and mutant cyanobacterial strains. Culture densities were measured at OD_750_ over five days. The error bars show the mean ± SE of four biological replicates. Letters denote significant differences (*p* < 0.05) between strains as determined by a repeated measures ANOVA followed by Tukey's honestly significant difference (HSD) tests. Abbreviations: SynO, *Synechocystis* Olive mutant; SynWT, *Synechocystis* (wild-type); TeWT, *T. elongatus* (wild-type).

To investigate if the expression of endogenous or heterologous PC had an impact on growth, the culture density of transconjugant strains with linker peptides (SynBAC2C1 and TeBACD) or without (SynBA and TeBA) was measured over five days and compared to that of SynO, WT *Synechocystis* and *T. elongatus* (Fig. 2). All transconjugant strains showed significantly increased growth rates compared to SynO. SynBAC2C1 and SynBA had similar growth rates, which was consistent with previous work that showed a *Synechocystis* Δ*cpcC2C1* mutant (equivalent to SynBA) with only one PC hexamer per rod is still sufficient for maximal light harvesting and biomass accumulation (Lea-Smith et al., 2014). SynBAC2C1 and SynBA had similar growth rates, while TeBACD performed slightly better than SynBAC2C1 and SynBA, and TeBA was marginally better than TeBACD. Nevertheless, growth rates in all transconjugant strains were reduced compared to WT *Synechocystis*. The growth rate of *T. elongatus* under optimal conditions at 45 °C was within the range previously reported for *Thermosynechococcaceae* and slightly better than that of SynO at 30 °C (Eberly and Ely, 2012; Klepacz-Smolka et al., 2020; Liang et al., 2018). Notably, *T. elongatus* failed to grow at 30 °C.

### 3.4 Te-PC forms a complex with the endogenous APC core

**Fig. 3:**
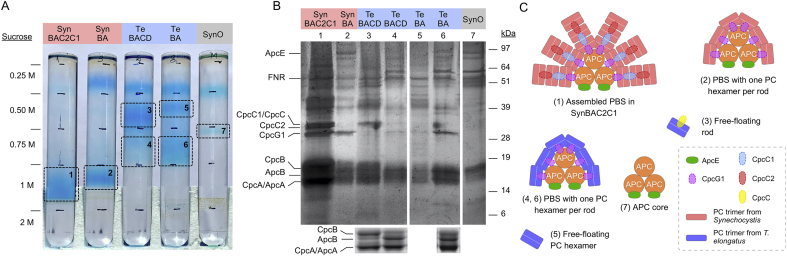
Complexes in phycobilisome extracts from Synechocystis Olive mutants transconjugated with endogenous or heterologous PC operons. (A) Sucrose step gradient of PBS extracts. Highlighted bands 1–7 were extracted for further analysis (B) Separation of the protein components in bands 1–7 by SDS-PAGE gel electrophoresis. Separation of the 14–19 kDa region of sucrose bands 3, 4 and 6 is shown below. The protein bands were visualized using silver staining. (C) Putative schematic structures of the PBS-associated complexes based on the components observed in bands 1–7. Abbreviations: APC, allophycocyanin; ApcA/ApcB, allophycocyanin ⍺- and β-subunits; ApcE, allophycocyanin core linker; CpcA/CpcB, phycocyanin ⍺- and β-subunits; CpcC/CpcC1/CpcC2, rod linker proteins; CpcG1, rod-core linker proteins; FNR, ferredoxin-NADP + oxidoreductase; PC, phycocyanin; SynO, *Synechocystis* Olive mutant; SynWT, *Synechocystis* (wild-type); TeWT, *T. elongatus* (wild-type).

Given the observed increases in growth for SynO transconjugants expressing Te-PC, we examined if Te-PC components might be incorporated into the endogenous PBS complex. PBS extracts from different transconjugant strains, including TeBACD and TeBA, were subjected to sucrose density gradient ultracentrifugation to separate complexes within the extracts (Fig. 3A). A total of seven bands were extracted, precipitated with ammonium sulfate and the components analysed by SDS-PAGE (Fig. 3B). Putative schematic structures of the six PBS-associated complexes based on the SDS-PAGE profile of each sucrose band are shown in Fig. 3C. The upper bands in the 0.25 M region consisted of free-floating PBPs and were not isolated (Lea-Smith et al., 2014; Ughy and Ajlani, 2004). SynBAC2C1 produced a single band in the 1.0 M region (band 1) that contained proteins typically associated with the PBS complex, including subunits of PC and APC, the rod-core linker protein CpcG1 and both rod linker proteins CpcC1 and CpcC2. The latter two linker proteins were absent from the band in SynBA (band 2), but the subunits of PC (i.e. CpcA and CpCB) and APC (i.e. ApcA and ApCB), and CpcG1 were still present, which suggested the assembly of a PBS complex with a single PC hexamer per rod attached to the APC core.

In contrast to SynBA and SynBAC2C1, two bands were produced in the sucrose density gradient ultracentrifugation for TeBACD and TeBA. For TeBACD there was a more abundant band in the 0.5 M region (band 3) and a fainter band in the 0.75 M region (band 4). Band 3 contained the heterologous rod linker protein CpcC but not CpcG1, suggesting that this complex consisted of a free-floating rod attached to one or two Te-PC hexamers. The fainter band 4 contained CpcG1, and thus indicated a slightly weaker capacity to form a complex similar to that in SynBA (band 2). In support of this hypothesis, further analysis of the 14–19 kDa region confirmed that band 3 primarily consisted of the CpcB/CpcA subunits of PC, whereas band 4 was enriched with ApcA/ApcB subunits that make up the APC core (Fig. 3B, bottom).

In TeBA, band 5 was located at a slightly higher position compared to band 3. The interactions between (⍺β)_3_/(⍺β)_3_ PC trimers are stronger in thermophilic species (Ferraro et al., 2020b), thus we hypothesised that band 5 consisted of a Te-PC hexamer pool (as in band 3) but with no CpcC rod linker present. In contrast, the protein composition of the more abundant lower band in TeBA (band 6) was nearly identical to that observed in SynBA (band 2) and included the CpcG1 rod-core linker and ApcE core membrane linker. Notably, the relative abundance of PC to APC subunits was higher in band 6 compared to band 4, which suggested that PBS complexes in TeBA have more Te-PC hexamers attached than in TeBACD (i.e. in the absence of the heterologous cpcC rod linker). Finally, in the absence of PC band 7 in SynO only contained the APC core and ApcE core membrane linker.

Through comparison of bands 4, 6 and 7, we hypothesised that the Te-PC hexamer in TeBACD and TeBA could interact with the endogenous CpcG1 rod-core linker to form a hybrid PBS complex with the endogenous APC core. Our results also suggested that the presence of the *T. elongatus* CpcC rod linker in TeBACD allowed for the formation of a Te-PC rod complex (band 3), but this could not attach to the endogenous APC core. Recent crystal structures of PBS complexes have shown that the N-terminus of the rod-core linker (CpcG) interacts with the C-terminus of the rod linker (CpcC) inside the first PC hexamer disc (Ma et al., 2020; Zhang et al., 2017). Therefore, the endogenous CpcG1 rod-core linker may be incompatible with the *T. elongatus* CpcC rod linker, which consequently limited the formation of hybrid PBS complexes in TeBACD. The latter hypothesis is also supported by the increased growth rates observed for TeBA compared to TeBACD (Fig. 2).

### 3.5 Te-PC transfers energy to PSII and PSI

**Fig. 4:**
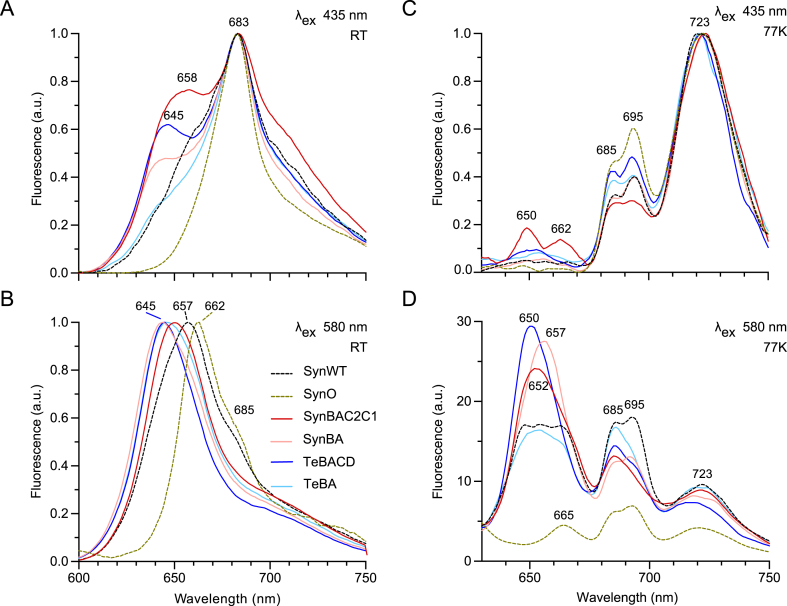
Room temperature and 77 K fluorescence spectroscopy of intact cyanobacterial cells. RT fluorescence of whole cells excited at (A) 435 nm and (B) 580 nm. Fluorescence was normalised at maximum chlorophyll fluorescence (683 nm) in A and at maximum PBS fluorescence in B. 77 K fluorescence of whole cells excited at (C) 435 nm and (D) 580 nm. Fluorescence was normalised at the maximal value in C and at the minimal value at 800 nm in D. The strains are colour-coded identically in A-D. Abbreviations: SynO, *Synechocystis* Olive mutant; SynWT, *Synechocystis* (wild-type). (For interpretation of the references to colour in this figure legend, the reader is referred to the Web version of this article.)

To investigate if light energy could be transferred from Te-PC through the endogenous APC core to PSII *in vivo*, we measured the steady-state fluorescence spectra of PSII and the PBS pool of whole *Synechocystis* cells at room temperature (RT) following excitation at 435 nm and 580 nm, respectively. The RT fluorescence spectrum of PSII in WT *Synechocystis* was characterised by a major peak at 683 nm attributed to PSII chlorophyll fluorescence and a smaller left shoulder typically associated with PC (Fig. 4A) (Stoitchkova et al., 2007). SynO lacked the left shoulder band due to absence of PC. In contrast, SynBAC2C1, SynBA and TeBACD showed prominent left shoulders between 645 and 660 nm, suggesting the presence of uncoupled PC in these strains. Notably, the left shoulder of TeBA was comparatively reduced, indicating that the excitation energy is more efficiently passed to the APC core. This might be linked to a more efficient coupling of Te-PC to the APC core in the absence of the heterologous CpcC rod linker, as supported by the associated sucrose gradient data (Fig. 3).

The RT fluorescence spectrum of the PBS pool in WT *Synechocystis* produced a major peak at 657 nm, which is associated with the combined emissions of PC and APC (Chen et al., 2015; Elanskaya et al., 2018), and a shoulder at 685 nm attributed to the APC terminal emitters and PSII (Fig. 4B). In the absence of PC and the presence of only the APC terminal emitters, the peak in SynO was shifted to 662 nm (Adir, 2005). Conversely, in all complemented strains the peak was shifted to 645–650 nm, again indicating the presence of uncoupled PC (Elanskaya et al., 2018). The peak shift in SynBAC2C1 to 650 nm was similar to that observed for a *Synechococcus* sp. PCC 7002 mutant lacking *cpcD*, suggesting the cap linker protein might have a similar effect on the integrity of the PBS complex in both species (de Lorimier et al., 1990). Notably, the right shoulder of SynBA and TeBA was shifted towards longer wavelengths by 3–4 nm compared to TeBACD, indicating a better energy transfer between PC and APC (David et al., 2014; Zhao et al., 1999). Together this data provided additional evidence that the energetic coupling of Te-PC to the APC core in TeBA was stronger than in TeBACD (Zhao et al., 1999).

Low temperature (77 K) steady-state fluorescence emission spectra were then analysed to further explore the energetic relationship between Te-PC and the endogenous photosystems (Lamb et al., 2018). Excitation at 435 nm produced three peaks for chlorophyll molecules in PSI (723 nm) and PSII (685 nm and 695 nm) (Fig. 4C) (Andrizhiyevskaya et al., 2005). Similar to the RT fluorescence data, SynBAC2C1 showed two peaks at 650 nm and 662 nm, suggesting a pool of energetically uncoupled PBPs. The fluorescence peaks at 695 nm and 723 nm were used to estimate the fluorescence peak ratio of PSII to PSI (Supplementary Table S2) (Elanskaya et al., 2018; Kana et al., 2014; Murakami, 1997). As expected for cyanobacteria, the PSII:PSI ratio was low in WT *Synechocystis* (0.4) (Vermaas, 2001). In contrast, the PSII:PSI ratio was higher in SynO (0.59) indicating an increased PSII pool relative to PSI. A comparable ratio was reported previously for the PC-deficient mutants PMB11 and CK (Ajlani et al., 1995; Collins et al., 2012) and reflects an adaptive mechanism to compensate for decreased capacity to reduce the plastoquinone pool (Vermaas, 2001). The ratio was restored to WT or near-WT levels (i.e. 0.29–0.46) in mutants complemented with either endogenous or heterologous PC. Thus, the expression of Te-PC in the *Synechocystis* Olive host was able to restore the photosystems to ratios normally observed in WT *Synechocystis*.

Excitation at 580 nm resulted in several emission peaks that are typically associated with PC (650 nm), APC (665 nm), the APC terminal emitters and PSII (685 nm), PSII (695 nm) and PSI (723 nm) (Fig. 4D). The spectra were further used to roughly estimate the energy transfer from PBSs (650–665 nm) to PSII (peak at 695 nm) and to PSI (peak at 723 nm) (Kana et al., 2014). Consistent with previous data for WT *Synechocystis*, the fluorescence peaks at 650 nm, 665 nm, 685 nm and 695 nm were of similar intensity, which indicated efficient energy transfer from the PBS to PSII and minimal fluorescence of individual PBPs or uncoupled PBSs (Ajlani and Vernotte, 1998; Ajlani et al., 1995; Elanskaya et al., 2018). As expected, SynO lacked a PC band and only a minor fluorescence peak at 665 nm from APC was observed due to weak energy absorption at 580 nm. In contrast, SynBAC2C1, SynBA and TeBACD showed three fluorescent peaks at 650 nm, 652 nm and 657 nm, respectively, from energetically uncoupled PC (Ajlani et al., 1995; Rakhimberdieva et al., 2007; Ranjbar Choubeh et al., 2018). Notably, in these strains the fluorescence at 685 nm and 695 nm was still significantly higher than SynO, suggesting that at least partial energy transfer to PSII was restored. Out of all strains tested, the levels of fluorescence at 650 nm and 685 nm in TeBA were most similar to WT *Synechocystis*, supporting our previous hypothesis that, in the absence of CpcC, Te-PC is able to be energetically coupled to the APC core. However, based on the fluorescence intensity at 695 nm, the efficiency of energy transfer to PSII in TeBA was still slightly lower than in WT *Synechocystis*.

Strains complemented with PC also showed evidence of energy transfer to PSI, as indicated by increased fluorescence at 723 nm compared to SynO, suggesting the capacity of heterologous PBS complexes to attach to the PSI complex. Similar results was previously observed in *Synechocystis* mutants that lacked the chromophorylated domain in ApcE, in which the PBS was unable to transfer energy to PSII (Elanskaya et al., 2018) and a similar emission spectrum was shown in isolated PBS-PSI complexes (Liu et al., 2013; Watanabe et al., 2014). However, we cannot preclude that this fluorescence signal is due to energy spillover from PSII due to the close proximity of these complexes on the thylakoid membrane (Bruce et al., 1989; Ranjbar Choubeh et al., 2018; Rast et al., 2019). Nevertheless, as for other wavelengths TeBA demonstrated fluorescence levels most similar to WT *Synechocystis*.

### 3.6 Te-PC content in transconjugants was similar to endogenous PC in *Synechocystis*

**Fig. 5:**
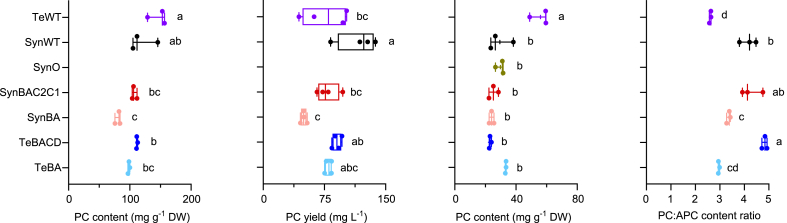
Phycocyanin and allophycocyanin in wild-type and mutant cyanobacterial strains. PC content, PC yield, allophycocyanin APC content and the PC:APC content ratio was measured after five days of growth (Fig. 2). Letters denote significant differences (*p* < 0.05) between strains as determined by a one-way ANOVA followed by Tukey's honestly significant difference (HSD) tests. Abbreviations: APC, allophycocyanin; PC, phycocyanin; SynO, *Synechocystis* Olive mutant; SynWT, *Synechocystis* (wild-type); TeWT, *T. elongatus* (wild-type).

The PC content per dry weight (DW) of SynBAC2C1 was similar to WT *Synechocystis*, while SynBA was reduced by 20% (Fig. 5). In contrast, the levels of Te-PC in TeBACD and TeBA were comparable to PC levels in WT *Synechocystis*. On average, the PC content per dry weight did not differ significantly between WT *T. elongatus* and WT *Synechocystis*. The mean yield in mg L^−1^ was higher for WT *Synechocystis*, but for both organisms there was considerable variation between replicates, which makes it difficult to identify which performed better. Importantly, the Te-PC yield in TeBACD (90 ± 5 mg L^−1^) was comparable to that in TeBA (80 ± 4 mg L^−1^), and in both of these organisms the yield was more consistent than in *T. elongatus* at its optimal conditions, due to the faster growth rates of the transconjugates. In contrast, the APC content in *T. elongatus* was nearly double that of WT *Synechocystis*. The latter result is likely due to a larger pentacylindrical APC core in *T. elongatus* and/or a larger number of PBS complexes on the thylakoid membrane, which could serve as a compensation mechanism for the shorter PC rods in this species.

Previous EM images have suggested that the APC core isolated from *T. elongatus* consists of three APC cylinders, as opposed to a pentacylindrical core in *T. vulcanus* (Barber et al., 2003). However, recently published high resolution structures of PBS complexes from red algae (Ma et al., 2020; Zhang et al., 2017) have provided further evidence that the number of conserved linker domains (Pfam00427) present in ApcE can be used to predict the structure of the APC core: two, three or four linker domains correspond to bi-, tri- or pentacylindrical APC cores, respectively (Arteni et al., 2009; Chang et al., 2015; David et al., 2014; Yamanaka et al., 1980). Accordingly, the presence of four linker domains in ApcE in *T. elongatus* strongly implies a pentacylindrical APC core.

As the APC core was not modified in the *Synechocystis* strains, the ratio of PC to APC could be used to estimate the PC accumulation per PBS (de Lorimier et al., 1992). The PC:APC ratio was lower in *T. elongatus* (2.6 ± 0.1) compared to *Synechocystis* (4.2 ± 0.3), which was expected as, in addition to a larger APC core, the PBS in *T. elongatus* contains only up to two PC hexamers per rod compared to up to three hexamers in *Synechocystis*. However, the ratio in *T. elongatus* was higher than that reported for the closely related strain *Thermosynechococcus vulcanus* (1.5 ± 0.2) (David et al., 2014). The latter differences in ratio may be due an underestimation of the APC content in *T. elongatu*s, as we only analysed the soluble PC-rich fraction. The interaction between the PBS and photosystems in thermophilic species is stronger and, thus, some of the APC might have pelleted in the non-soluble thylakoid membrane fraction (Kana et al., 2014). In the transconjugated strain TeBACD, the presence of the CpcC rod linker correlated with a similar PC:APC ratio to that of WT *Synechocystis* and SynBAC2C1, which both expressed two rod linkers (Fig. 5). In contrast, the PC:APC ratio was reduced by 30% in the linkerless strains SynBA and TeBA. Together, these results suggested that the absence of rod linker peptides led to a reduced accumulation of PC per APC in SynBA and TeBA.

### 3.7 The thermostability of Te-PC extracted from *Synechocystis* is similar to that from *T. elongatus*

A temperature gradient fluorescence assay was used to compare the thermostability of PC extracted from WT *Synechocystis*, *T. elongatus* and the transconjugant strains. The fluorescent properties of PC are linked to the conformational rigidity of the PCB chromophores within the (⍺β) monomer and higher order (⍺β)_3_ trimer complex. Heat-based denaturation of PC relaxes the tertiary protein complex structure, leading to an increase in conformational freedom of the PCBs and a reduction in fluorescence. Fluorescence spectroscopy provides a highly sensitive method of measuring PC stability as even small changes in temperature (e.g. >0.5 °C) can affect PCB rigidity.

**Fig. 6:**
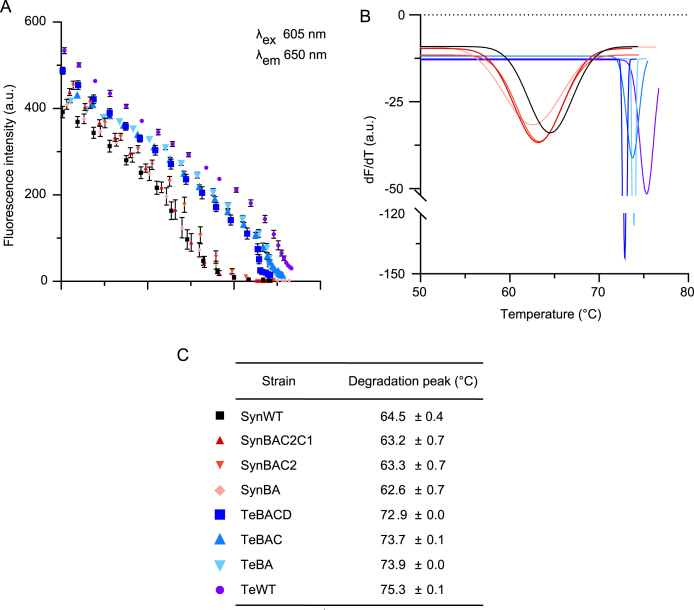
Thermostability of extracted PC measured using a temperature gradient fluorescence assay. (A) Temperature dependence of PC fluorescence monitored at 650 nm following excitation at 605 nm. Sample data between 50 °C and 80 °C is shown. Values are the mean ± SE of three technical replicates. (B) Gaussian curves fitted to the first derivatives of the degradation profiles in A. The location of each peak represents the temperature at which the maximal rate of PC degradation was reached. (C) Temperatures correspond to the degradation peak of PC for each line. The mean ± SE for each temperature is based on Gaussian fits of three technical replicates. The strains are colour-coded identically in A, B and C. Abbreviations: SynWT, *Synechocystis* (wild-type); TeWT, *T. elongatus* (wild-type). (For interpretation of the references to colour in this figure legend, the reader is referred to the Web version of this article.)

The fluorescence intensity of PC at 650 nm decreased for all samples as the temperature increased (Fig. 6A), which matched the expected trend for heat-induced PC degradation (Eriksen, 2008). Samples from strains producing endogenous *Synechocystis* PC showed a similar fluorescence degradation pattern, with a steady decline until *ca*. 60 °C followed by a sharp drop leading to a ‘degradation peak’ (Fig. 6A and B), which corresponds to the temperature at which PC is denatured. The average degradation peak for PC extracted from WT *Synechocystis* and transconjugant strains expressing native PC was 63.4 ± 0.4 °C (Fig. 6C). In contrast, Te-PC extracted from TeBACD, TeBAC, TeBA was more stable and comparable to *T. elongatus*, with an average degradation peak at 73.9 ± 0.5 °C.

### 3.8 Te-PC can be purified from *Synechocystis* using heat treatment

**Fig. 7:**
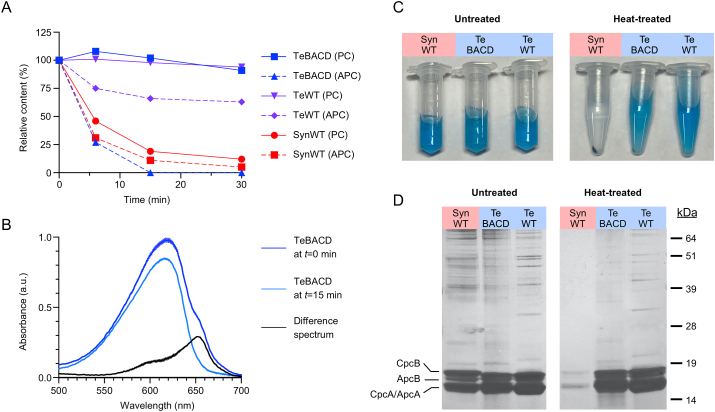
Purification of phycocyanin using heat treatment. (A) Analysis of the PC and APC contents in heat-treated PBS extracts after 6, 15 and 30 min of incubation at 60 °C expressed as a percentage of the contents (g L^−1^) in untreated control. Values are the mean ± SD of three technical replicates. (B) Absorption spectra of PC extracts from TeBACD in (A) before and after 15 min heat treatment, and the difference spectrum thereof. C) Images of tubes containing PC extracts and (D) Silver-stained SDS-PAGE gel of samples in (C). See Fig. 1 for abbrevation details.

As Te-PC from *Synechocystis* showed a similar stability compared to *T. elongatus*, we investigated if a short heat treatment of the PC extract following cell disruption and centrifugation could be used as a rapid and low-cost method to purify Te-PC from endogenous APC (APC is typically considered a contaminant when purifying PC) (El-Mohsnawy and Abu-Khudir, 2020; Sun et al., 2012; Zhang and Chen, 1999). We measured the change in PBP content (i.e. PC and APC) in the PC extracts from WT *Synechocystis*, TeBACD and *T. elongatus* during incubation at 60 °C over 30 min. The contents of PC and APC in WT *Synechocystis* dropped by 54% and 69%, respectively, of the initial values within 6 min of incubation, and declined further to 12% and 5% after 30 min (Fig. 7A). In contrast, PC and APC contents in *T. elongatus* remained at 94% and 63%, respectively, after the 30 min incubation. Similarly to *T. elongatus*, the PC content in TeBACD remained over 90% of the initial value. However, the APC content in TeBACD decreased sharply after 6 min of incubation. We observed that heat treatment for 15 min was sufficient to reduce APC content below detectable levels. Further analysis of the absorption difference spectra of PC extracts before and after 15 min of heat treatment showed a substantial decrease in absorbance between 500 nm and 750 nm, with a peak at 652 nm corresponding to the absorption of APC trimers (Fig. 7B) (MacColl, 2004). A similar difference spectrum was also observed in PBS isolated from *Synechocystis* mutant lacking *apcAB* genes (Niedzwiedzki et al., 2019).

Following 15 min of the heat treatment, PC extracts from TeBACD and *T. elongatus* remained blue and soluble (Fig. 7C). In contrast, PC extracts from WT *Synechocystis* showed a loss of colour and protein precipitation, which could be pelleted by centrifugation (Fig. 7C). Subsequent separation of the heat-treated protein samples on a Coomasie-stained SDS-PAGE gel confirmed a decrease in background soluble protein in PC extracts from WT *Synechocystis* (Fig. 7D). In contrast, heat treatment had only a minor impact on PC extracts from *T. elongatus*. In extracts from TeBACD, the thermostable ⍺- and β-subunits of Te-PC remained largely unchanged compared to untreated samples, while endogenous APC and background soluble protein were similarly reduced, as in WT *Synechocystis*. Thus, our results show that heat treatment can be used as an effective, low-cost method to purify Te-PC from endogenous APC and the soluble protein pool when expressed in a mesophilic cyanobacterium.

### 3.9 Production of Te-PC in *Synechocystis* is more energy efficient than in *T. elongatus*

To calculate the energetic cost of producing Te-PC at 30 °C in *Synechocystis* compared to *T. elongatus* at 45 °C, a heat and mass transfer model was generated based on a hypothetical cylindrical bubble column photobioreactor (PBR) with a fixed diameter (20 cm) for optimal light penetration (Lea-Smith et al., 2014) (Supplementary Fig. S3A) (see Supplementary materials and methods). The model was used to estimate the energy input in Watts (W) required to offset the heat loss from the PBR and maintain an appropriate temperature (i.e. for growth at 30 °C or 45 °C) during continuous production of 1 g of PC per day. The biomass productivity during exponential stage (mg L^−1^ d^−1^) was calculated from the growth analysis data (Fig. 2A) and combined with the PC content (mg g^−1^ DW) (Fig. 5) to calculate the PC productivity (mg L^−1^ d^−1^) of different strains (Table 1). The results highlighted the important contributions of both culture growth rate and PC content to PC productivity (Eriksen, 2008). The lower biomass productivity of *T. elongatus* at 45 °C compared to TeBA and TeBACD at 30 °C was offset by a higher PC content, which resulted in a similar PC productivity between these strains. Therefore, production of 1 g of Te-PC per day in *T. elongatus* or strains TeBA and TeBACD would require PBRs of comparable volumes (52–57 L) (Table 1). However, the heat losses for culturing *T. elongatus* at 45 °C were on average 3.4-fold greater than for TeBA and TeBACD at 30 °C. For example, over a 21-day culture growth period 63 kWh would be saved by growing TeBACD for the production of 1 g of Te-PC compared to in *T. elongatus*. The difference in energy consumption at 30 °C and 45 °C increases dramatically when production is upscaled to 100,000 L, reaching 93,170 kWh (Supplementary Fig. S3B). According to the US Environmental Protection Agency greenhouse gas equivalencies calculator, a PC production plant with a 100,000 L capacity that runs 12 cycles per year would save 791 metric tons in CO_2_ emissions, which is the equivalent to the annual outputs of 171 petroleum-fuelled passenger vehicles (i.e. 2-axle 4-tire) (EPA, 2019). Therefore, expression of Te-PC in *Synechocystis* strains could provide significant energy and carbon savings during industrial cultivation while maintaining a comparable Te-PC productivity to *T. elongatus*. Further optimisation of the PBR setup and growth conditions for *Synechocystis* (e.g. light, growth medium, aeration), as has been developed for *A. platensis* (Chen et al., 2010, 2013a; Lima et al., 2018; Rio-Chanona et al., 2015), could further increase PC productivity and energy efficiencies.

**Table 1:** Biomass and phycocyanin yields, and heat loss estimations for a hypothetical photobioreactor.

Strain	Biomass productivity ^*a*^ (mg L^−1^ d^−1^)	PC productivity ^*b*^ (mg L^−1^ d^−1^)	PBR volume ^*c*^ (L) for 1 g PC d^−1^	Heat loss at 30 °C ^*d*^ (W)	Heat loss at 45 °C ^*d*^ (W)
*Synechocystis* (WT)	206	24.8	40.2	39.2	–
SynBAC2C1	154	16.5	60.4	56.2	–
SynBA	128	10.3	96.7	86.3	–
TeBACD	173	19.4	51.6	48.8	–
TeBA	177	17.4	57.3	53.5	–
*T. elongatus*	120	17.5	57.0	–	173.1

^*a*^ Biomass productivity was calculated by measuring the difference in biomass concentration (mg L^−1^) at the start and end of the exponential stage and dividing by the number of days between measurements as described in Materials and methods. ^*b*^ PC productivity was calculated by multiplying the biomass productivity by the PC content of each strain. ^*c*^ The volume of the PBR was calculated by dividing the target PC productivity (1 g L^−1^ d^−1^) by the PC productivity of each strain. ^*d*^ Heat loss was calculated for the production of 1 g of PC per day according to the heat and mass transfer model described in Supplementary materials and methods.

## 4 Conclusions

Here we have demonstrated for the first time that a heterologous, thermostable phycobiliprotein can be expressed in a mesophilic species and produced at comparable yields compared to the host species, while significantly reducing energy consumption and carbon emissions. Our PC production platform also offers improvements in downstream processing by using a cost-efficient heat treatment to purify the final product. Importantly, our results indicate that Te-PC forms a functional hybrid PBS complex with the APC core *in vivo* in *Synechocystis*, which allows for energy transfer to the photosystems. The hybrid PBS also appeared to be more stable at increased temperatures compared to the WT PBS, suggesting a stronger interaction of the APC core with Te-PC than with endogenous PC. However, we found limited evidence of interaction when Te-PC was co-expressed with the heterologous rod linker CpcC, suggesting that the endogenous CpcG1 rod-core linker is not compatible with CpcC. Thus, the rod length of the hybrid PBS is currently limited to a single Te-PC hexamer. This incompatibility could be overcome by generating a fusion CpcG or CpcC linker to maintain the native linker-to-linker interface inside the rod. Engineering synthetic linkers could open up the possibility of further increasing the length of PC rods and/or expanding the light absorption region of the PBS by incorporating additional PBPs with maximal absorption below 620 nm, such as phycoerythrin (Six et al., 2007). Furthermore, engineering functional hybrid PBS complexes could improve the growth performances of promising cyanobacterial and algal strains for biotechnology and biophotovoltaic applications.

## CRediT authorship contribution statement

**Anton Puzorjov:** Conceptualization, Writing – original draft, Investigation, Methodology, Formal analysis, Visualization. **Katherine E. Dunn:** Writing – review & editing, Resources. **Alistair J. McCormick:** Supervision, Writing – original draft, Funding acquisition, Resources.

## Declaration of competing interest

There are no competing interests to declare.
