# Supplementary material for: Production of thermostable phycocyanin in a mesophilic cyanobacterium
Source: Metab Eng Commun. 2021 Jun 2;13:e00175. doi: 10.1016/j.mec.2021.e00175 (PMC8209669; doi:10.1016/j.mec.2021.e00175)
Supplement: Supplementary Figures and Tables [file mmc2.pdf]

Engineering the production of thermostable phycocyanin in a mesophilic cyanobacterium

Anton Puzorjov[1], Katherine E. Dunn[2], Alistair J. McCormick[1,†]

[1]SynthSys & Institute of Molecular Plant Sciences, School of Biological Sciences, University of Edinburgh, Edinburgh, EH9 3BF, UK

[2] Institute for Bioengineering, School of Engineering, University of Edinburgh, Edinburgh, EH9 3DW, UK

Supplementary information

Supplementary Figure S1. Transconjugant *Synechocystis* Olive mutants expressing endogenous or heterologous phycocyanin.

Supplementary Figure S2. Comparison of rod linker peptides in *Synechocystis* and *T. elongatus*.

Supplementary Figure S3. Modelled photobioreactor design and estimated energy consumption at different temperatures.

Supplementary Table S1. List of primers used in this study.

Supplementary Table S2. Ratio of photosystem II to photosystem I.

Supplementary Table S3. Air properties at different film temperatures used to calculate the Grashof number.

Supplementary materials and methods.

Supplementary Information S1. Sequence maps (.gb files) of plasmid vectors carrying operons in Figure 1B. See .zip file.

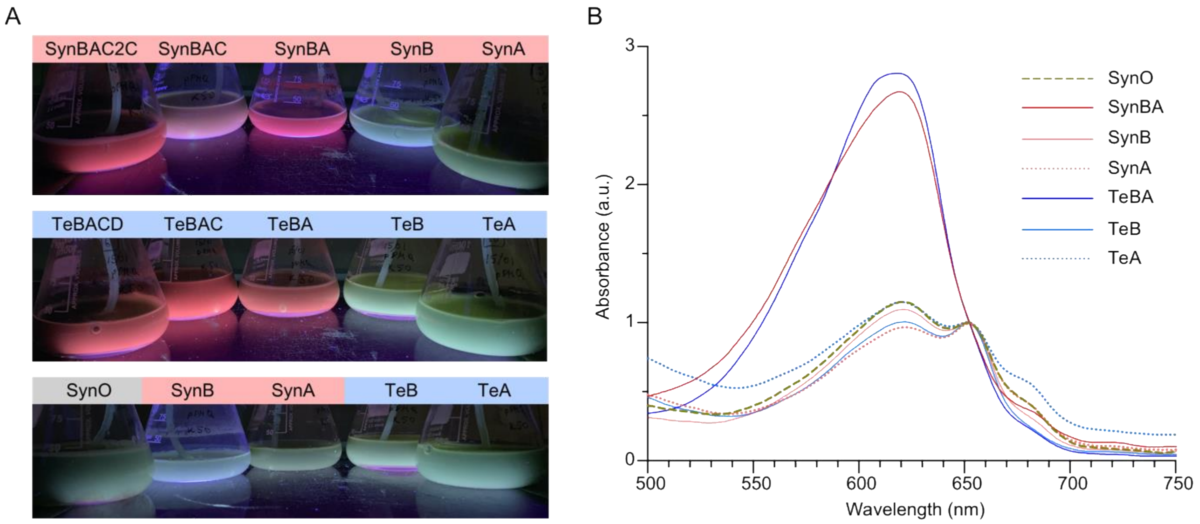

Supplementary Figure S1. Transconjugant *Synechocystis* Olive mutants expressing endogenous or heterologous phycocyanin. (A) Cultures in UV light (312 nm). (Seppälä et al., 2007) (B) Absorption spectra of the cell extracts showing the lack of a typical PC peak at 620 nm in transconjugants containing individual PC α- and β-subunits. The absorption was normalised to APC peak at 652 nm.

A

```
sp|P73204|PYR2_SYNY3      MTSLVSAQRLGIVAVDEAIPLELRSRSTEEEVDAVILAVYRQVLGNDHLMSQERLTSAES 60
sp|P73203|PYR1_SYNY3      MAITTAASRLGVAPYNESRPVELRPDFSLDDAKMVIRAVYRQVLGNDYIMDSERLKGAES 60
sp|P50034|PYR1_THEEB      MAITAAASRLGTSAFSDAPPVELRANWSEEDLETVIRAVYRQVLGNDYVMASERLVSAES 60
                          *:  .:*.***    .:: *:***   : :: . ** **********::* .*** .***

sp|P73204|PYR2_SYNY3      LLRGREISVRDFVRAVALSEVYRQKFFHSNPQNRFIELNYKHLLGRAPYDQSEIAFHTDL 120
sp|P73203|PYR1_SYNY3      LLTNGSISVREFVRTVAKSELYKKKFLYNNFQTRVIELNYKHLLGRAPFSEDEVIFHLDL 120
sp|P50034|PYR1_THEEB      LLRNGKITVREFVRAVAKSELYKEKFLYGNFQTRVIELNYKHLLGRAPYDESEVIFHLDL 120
                          ** . .*:**:***:** **:*::**::.* *.*.*************:.:.*: ** **

sp|P73204|PYR2_SYNY3      YHQGGYEAEINSYIDSVEYTENFGDWVVPYFRGFATQRNQKTVGFSRSFQVYRGYATSDR 180
sp|P73203|PYR1_SYNY3      YENQGFDADIDSYIDSVEYQENFGENIVPYYR-FNNQVGDRTVGFTRMFRLYRGYANSDR 179
sp|P50034|PYR1_THEEB      YENEGFDADIDSYIDSPEYTNSFGDWVVPYYRGFNTQPGQKTVGFNRIFRLYRGYANSDR 180
                          *.: *::*:*:***** ** :.**: :***:* * .* .::****.* *::*****.***

sp|P73204|PYR2_SYNY3      SQGNGSRSRLTRELARNTASPVYAGSTAE----------------SLRGT-SAGSRNQMY 223
sp|P73203|PYR1_SYNY3      SQLERSSSRLATELGQNTVSAIVGPSGSNAGWAYRPSRAGNTPAKALGGTVPFGQASKLF 239
sp|P50034|PYR1_THEEB      AQAEGSMSRLARDLATNRANTVVPPSNSDTAFAYYTPSADVPPRACLGGS--FGESGRVY 238
                          :* : * ***: :*. * .. :   * ::                .* *:   *. .:::

sp|P73204|PYR2_SYNY3      RLQVIQGAAPGRGTRVRRGKAEYLVSYDNLSAKLQQINRQGDTVTMISLA--- 273
sp|P73203|PYR1_SYNY3      RVEITAISAPGY-PKVRRSNKAVIVPFEQLNQTLQQINRLGGKVASITPASLS 291
sp|P50034|PYR1_THEEB      RIEVAGIRQPGY-PGVRRSSTAFLVPYEQLSAKMQQLQRTGARIISVNPA--- 287
                          *:::     **    ***..   :* :::*. .:**::* *  :  :. *
```

B

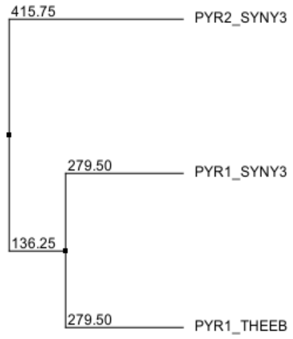

Supplementary Figure S2. Comparison of rod linker peptides in *Synechocystis* and *T. elongatus*. (A) Multiple sequence alignment of CpcC1 (PYR1_SYNY3) and CpcC2 (PYR2_SYNY3) from *Synechocystis*, and CpcC (PYR1_THEEB) from *T. elongatus* using Clustal Omega (v1.2.2). Entry names on UniProt are noted in the brackets. (B) A phylogenetic tree was constructed using distance matrices determined from aggregate BLOSUM 62 score using the Average Distance (UPGMA) algorithm. The distance values above the branches were calculated in JalView (v2.11).

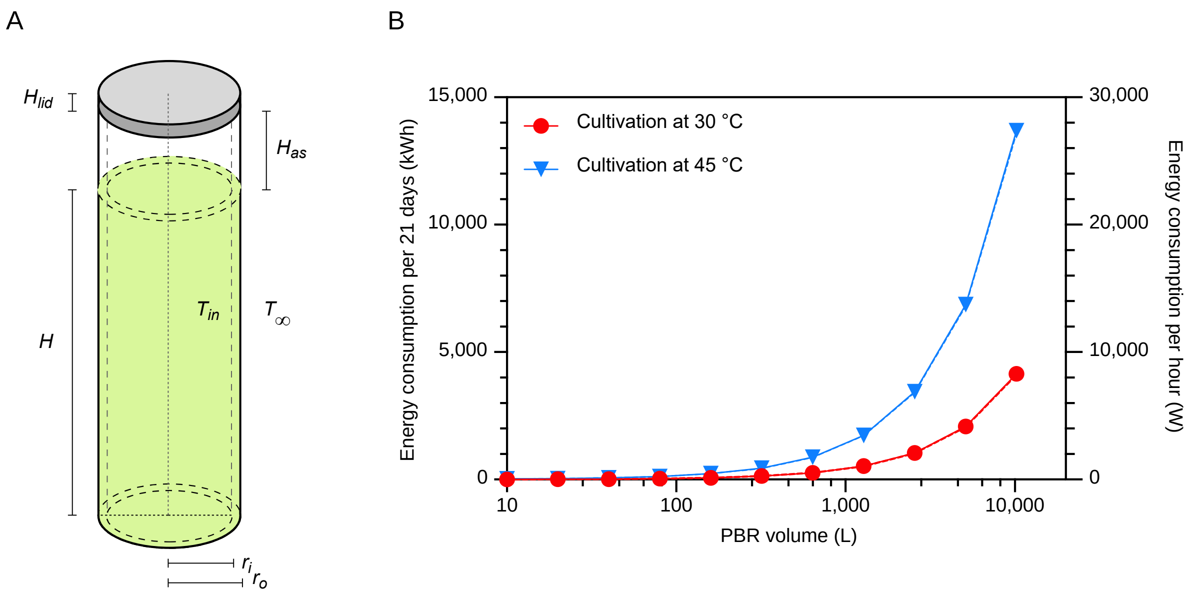

Supplementary Figure S3. Modelled photobioreactor design and estimated energy consumption at different temperatures. (A) A schematic diagram of the photobioreactor (PBR) model. (B) Model output showing the energy consuption per hour (right y-axis) and per 21-day cultivation cycle (left y-axis) required to maintain a glass PBR with a fixed outer diameter (20 cm) at a growth temperature of 30 °C or 45 °C.

Supplementary Table S1. List of primers used in this study.

ID	Primer sequence (5′ -> 3′)[a]
Syn cpcA F	cagtgaagacataATGAAAACCCCTTTAACTGAAGCCG
Syn cpcA R	cagtgaagacataagcCTAGCTCAGAGCATTGATGGCG
Syn cpcB F	cagtgaagacataATGTTCGACGTATTCACTCGGG
Syn cpcB-BsaI F	ctcaatggaagactcGGACTCCGTGAAACCTACGTTGCC
Syn cpcB-BsaI R	ctcaatggaagactcGTCCGTTCAAGCAACGATCTTCTAGAACG
Syn cpcB R	cagtgaagacataagcCTAGGCTACGGCAGCAGC
Syn cpcC1 R	cagtgaagacataagcCTAGCTCAAGCTAGCCGGG
Syn cpcC2 R	cagtgaagacataagcCTAGGCAAGGGAAATCATTGTGACC
Te cpcA F	cagtgaagacataATGAAAACGCCGATTACTGAAGCT
Te cpcA R	cagtgaagacataagcTAGCTGAGGGCGTTAATGGCG
Te cpcB F	cagtgaagacataATGCTAGATGCATTTGCCAAAGTTGT
Te cpcB R	cagtgaagacataagcTTAGGCAACAGCGGCAGC
Te cpcC R	cagtgaagacataagcCTAGGCAGGATTGACACTGATAATGC
Te cpcD R	cagtgaagacataagcCTAGGAGGCAACCACTGGAGT

[a] BpiI recognition site is coloured in blue, the overhang is coloured in red and the ORF sequence is shown in uppercase letters.

Supplementary Table S2. Ratio of photosystem II to photosystem I. Following excitation at 435 nm, the relative 77K fluorescence intensity at 695 nm and 723 nm was used to estimate the ratio of PSII to PSI.

Strain	PSII/PSI ratio
SynWT	0.40
SynO	0.59
SynBAC2C1	0.29
SynBA	0.41
TeBACD	0.46
TeBA	0.40

Supplementary materials and methods

Calculation of heat loss from a photobioreactor

To calculate the energy input required maintain a constant temperature inside of a cylindrical bubble column photobioreactor (PBR)(Sánchez Mirón et al., 2004; Sánchez Mirón et al., 2000), a heat and mass transfer model was composed as described below. By definition, to maintain the constant temperature of the system, the heat input must be equal to the heat lost by the system to the surrounding environment. The model of the heat loss from the PBR is based on the following assumptions:

(1) the contents of the PBR are well mixed (i.e. the internal forced convection is negligible),
(2) the temperature of the outer glass surface of the PBR is equal to the temperature of the culture,
(3) surface radiation is negligible,
(4) the surrounding room serves as a heat sink,
(5) natural convection occurs around the PBR,
(6) the height (*H*) of the culture column inside the PBR depends on the culture volume with a fixed diameter (*d* = 20 cm) (Sánchez Mirón et al., 2004; Sánchez Mirón et al., 2000),
(7) the curvature effect of the PBR is negligible (i.e. the convection from the sides of the PBR can be calculated as convection along a vertical flat wall),
(8) the heat originated from the LED light source or generated by the cyanobacterial metabolism is not accounted for.

The heat from the liquid culture inside of the PBR (**Fig. S3**) is lost to the side walls, top and the bottom of the PBR vessel due to conduction, which is further lost to the outside air though convection. The steady state of heat transfer (q_{tot}) from the liquid culture was calculated using the following equation (Incropera et al., 2007):

$$\boldsymbol{q_{tot} = T_{in} - T_{\infty} \left(\frac{1}{R_{side}} + \frac{1}{R_{top}} + \frac{1}{R_{bottom}}\right)}$$

, where T_{in} is the temperature inside the bioreactor (30 °C for *Synechocystis* strains and 45 °C for *T. elongatus*), T_{∞} is the surrounding bulk room temperature (20 °C) and R_{side}, R_{bottom} and R_{top} are the thermal resistances of the side wall, bottom and top, respectively.

Assuming the internal convection is negligible, the thermal resistance of the side walls is the sum of individual thermal resistances of the radial conduction through the side wall and natural convection of the air (Incropera et al., 2007):

$$R_{side} = \frac{\ln(\frac{r_o}{r_i})}{k_g H 2\pi} + \frac{1}{h_{side} H 2\pi r_o}$$

, where r_i is the inner radius of the PBR, r_o is the outer radius of the PBR which is equal to the sum of r_i and of the PBR wall thickness (0.0015 m), H is the height of the culture column in PBR, k_g is the thermal conductivity of glass (1.4 W m^{-1} K^{-1}) (Incropera et al., 2007), h_{side} is the convective heat transfer coefficient of air.

The thermal resistance through the top PBR is the sum of individual thermal resistances of the air space cavity, the top lid and the natural convection of the air (Incropera et al., 2007):

$$R_{top} = \frac{1}{h_{as}\pi r_i^2} + \frac{H_{lid}}{k_{lid}\pi r_i^2} + \frac{1}{h_{top}\pi r_o^2}$$

where k_{lid} is the thermal conductivity of lid made of acrylonitrile butadiene styrene polymer (0.2 W m^{-1} K^{-1}) (Sonsalla et al., 2018) and h_{as} and h_{top} are the convective heat transfer coefficient of air in the air space cavity and on top of the lid, respectively.

The thermal resistance through the bottom of the PBR is the sum of individual thermal resistances of the conduction through the bottom glass and natural convection of the air (Incropera et al., 2007):

$$R_{bottom} = \frac{r_o - r_i}{\pi r_i^2 k_g} + \frac{1}{\pi r_o^2 h_{bottom}}$$

, where h_{bottom} is the convective heat transfer coefficient of the air.

Determination of the convective heat transfer coefficients of air

The convective heat transfer coefficients were calculated as follows (Incropera et al., 2007):

$$h_{side} = \frac{k_{air} Nu_{side}}{H}$$

$$h_{as} = \frac{k_{air} Nu_{as}}{H_{as}}$$

$$h_{top} = \frac{k_{air} Nu_{top}}{L}$$

$$h_{bottom} = \frac{k_{air} Nu_{bottom}}{L}$$

, where k_{air} is the thermal conductivity of air and H_{as} are the height of the culture column in the PBR and the air space, respectively. L is the characteristic length defined as the ratio between the surface of the area ($2\pi r_o^2$) and the perimeter ($2\pi r_o$). Nu_{side}, Nu_{as}, Nu_{top} and Nu_{bottom} are the Nusselt numbers calculated for the vertical flat wall, horizontal cavity heated from below, the upper and the lower surface of heated plate, respectively, and were calculated as follows (Incropera et al., 2007):

$$Nu_{side} = \left\{0.825 + \frac{0.387Ra_x^{1/6}}{(1 + (0.492/Pr^{9/16})^{8/27}}\right\}^{\mathbf{2}}$$

$$Nu_{as} = 0.069Ra_x^{1/3}Pr^{0.074}$$

$$Nu_{top} = 0.54Ra_x^{1/4}$$

$$Nu_{bottom} = 0.27Ra_x^{1/4}$$

, where Ra_x is the Rayleigh number, which is the product of Grashof (Gr) and Prandtl (Pr) numbers (Incropera et al., 2007):

$$Ra_x = Gr_x Pr$$

The Grashof number was calculated using properties of the air at the film temperature (T_f, **Table S3**), which the average of the surface and the surrounding bulk temperatures, using the following equation (Incropera et al., 2007):

$$Gr_x = \frac{g\beta(T_s - T_\infty)x^3}{v^2}$$

, where x is either the height of the culture column inside PBR (H), the inner ($2r_i$) or the outer diameter ($2r_o$) of the PBR, depending on whether the Grashof number is calculated for Nu_{side}, Nu_{as} or $Nu_{top/bottom}$ respectively and T_s is the surface temperature (~culture temperature). Other parameters are described in **Table S3**.

Supplementary Table S3. Air properties at different film temperatures used to calculate the Grashof number. The values of the variables were taken from Engineering ToolBox (Engineeringtoolbox.com, 2021).

Variable	T_f = 25 °C	T_f = 32.5 °C
Thermal expansion coefficient (β, K^{-1})	3.38 x 10^{-3}	3.21 x 10^{-3}
Gravity (g, m s^{-2})	9.8	9.8
Kinematic viscosity (v, m^2 s^{-1})	15.52 x 10^{-6}	16.22 x 10^{-6}
Thermal conductivity of air (K_{air}, W m^{-1} K^{-1})	26.24 x 10^{-3}	26.8 x 10^{-3}
Prandtl number	0.707	0.706

Supplementary References

Incropera, F. P., Lavine, A. S., Bergman, T. L., DeWitt, D. P., 2007. Fundamentals of heat and mass transfer. Wiley.

Sánchez Mirón, A., Cerón García, M. C., García Camacho, F., Molina Grima, E., Chisti, Y., 2004. Mixing in Bubble Column and Airlift Reactors. Chemical Engineering Research and Design. 82**,** 1367-1374.

Sánchez Mirón, A., García Camacho, F., Contreras Gómez, A., Grima, E. M., Chisti, Y., 2000. Bubble-column and airlift photobioreactors for algal culture. AIChE Journal. 46**,** 1872-1887.

Seppälä, J., Ylöstalo, P., Kaitala, S., Hällfors, S., Raateoja, M., Maunula, P., 2007. Ship-of-opportunity based phycocyanin fluorescence monitoring of the filamentous cyanobacteria bloom dynamics in the Baltic Sea. Estuarine, Coastal and Shelf Science. 73**,** 489-500.

Sonsalla, T., Moore, A. L., Meng, W. J., Radadia, A. D., Weiss, L., 2018. 3-D printer settings effects on the thermal conductivity of acrylonitrile butadiene styrene (ABS). Polymer Testing. 70**,** 389-395.
